# To What Degree Does Limb Spasticity Affect Motor Performance in Para-Footballers With Cerebral Palsy?

**DOI:** 10.3389/fphys.2021.807853

**Published:** 2022-01-24

**Authors:** Alba Roldan, Matías Henríquez, Aitor Iturricastillo, Daniel Castillo, Javier Yanci, Raul Reina

**Affiliations:** ^1^Sports Research Centre, Department of Sport Sciences, Miguel Hernández University, Elche, Spain; ^2^Sports and Physical Exercise Research Group (GIKAFIT), Department of Physical Education and Sport, Faculty of Education and Sport, University of the Basque Country (UPV/EHU), Vitoria-Gasteiz, Spain; ^3^Faculty of Education, University of Valladolid, Soria, Spain

**Keywords:** soccer, classification, para-athlete, para-sport, Paralympic, brain impairment

## Abstract

Spasticity is considered a contributor to hypertonia, frequently presented in people with cerebral palsy (CP), affecting muscle function and motor activities. In CP football, the classification system determines that this impairment is eligible for competitive para-sports due to the impact on activity limitation and sports performance. However, the relationship between this feature (i.e., spastic hypertonia) and performance determinants has not been explored yet. This study aimed to assess the association of clinical spasticity measurements with the performance of sport-specific tests used for classification purposes. Sixty-nine international footballers with CP voluntarily participated in this study. The Australian Spasticity Assessment Scale was used to measure spasticity in lower limbs muscle groups and activity limitation tests were conducted considering dynamic balance, coordination, vertical and horizontal jumps, acceleration, and change of direction ability. Low-to-moderate negative significant associations were found between the hip spasticity and measures of dynamic balance and dominant unipedal horizontal jump capacity. Additionally, moderate associations were reported between the knee spasticity and the non-dominant unipedal horizontal jump capacity and the change of direction actions with the ball. The ankle spasticity score reported small to moderate associations with the change of direction assessment without the ball and bipedal and dominant unipedal horizontal jump capability. Finally, the total spasticity score only presented a significant association with horizontal jump performance. This is a novel study that provides evidence of the associations between an eligible neural impairment and relevant specific measures of activity limitation tests. These results suggest that the amount of spasticity according to each evaluated joint muscle group of the lower limbs presents a low-to-moderate significant relationship with determined measures of dynamic balance, coordination, horizontal jump, acceleration, and change of direction ability with and without the ball in international-level CP footballers. Further studies are necessary to elucidate the real contribution of neural and non-neural impairments related to hypertonia on fundamental sport-specific motor skills of para-footballers with CP.

## Introduction

In para-sport, evidence-based grading is mandatory to develop a competition system that ensures that outcomes are based on a causal relationship between eligibility and the impact of the eligible impairment on sports performance (Tweedy and Vanlandewijck, 2011). Current advances in the understanding of Paralympic classification identify the necessity to determine the strength of association between valid, reliable, and ratio-scaled measurements of eligible impairments and performance in sport-specific activities (Tweedy and Vanlandewijck, 2011; Tweedy et al., 2014). However, the development of evidence-based classification research for para-athletes with eligible impairments of hypertonia, athetosis or ataxia (i.e., those with cerebral palsy [CP] and other related neurological conditions) is still a challenge in para-sports (Tweedy and Vanlandewijck, 2011; Tweedy et al., 2014, 2016, 2018). Para-athletes with CP are usually eligible due to the presence of a minimum impairment criteria concerning hypertonia, ataxia, or athetosis, which is evaluated with clinical measures to determine the severity of the involvement and its impact when performing sports-specific skills (Connick et al., 2018). However, a recent study has suggested that classification research in para-athletes with CP should consider impairment-specific and performance relationships, avoiding lumping all three impairments into a unique group (Reina et al., 2020b).

Among the three eligible impairments, hypertonia, or related impairments (i.e., spastic syndromes) are the most common type of CP, resulting from the injury of the upper motor neurons and possibly the loss of descending inhibitory input, thereby altering muscle tone, which intensifies with movement velocity (Odding et al., 2006; Graham et al., 2016). The upper motor neuron is the first-order neuron in the central nervous system, which contains axons that carry impulses responsible for movement providing a direct pathway between the cerebral cortex through the corticospinal tract synapsing with a lower motor neuron in the spinal cord (Menon and Vucic, 2021). In people with CP, the upper motor neuron lesion occurs due to a brain injury of non-progressive characteristics in the developing fetal or infant brain, causing a group of permanent disorders affecting movement, posture and causing activity limitation (Graham et al., 2016). The consequences of the impairments in CP can be of neural-related mechanism origin, as increased pathological tone (i.e., spasticity), hyperreflexia, and other recognized clinical features (de Gooijer-van de Groep et al., 2013). On the other hand, non-neural origins are referred to the altered muscle properties such as tissue viscoelastic and stiffness which are often altered in cerebral palsy (Bar-On et al., 2015).

Specifically, spasticity is a clinical phenomenon thought to be the result of a hyper-excitable position and velocity-dependent stretch reflexes, resulting in a relevant contributor to muscle hypertonia with neural-physiological adaptations that shapes motor function over time (Bar-On et al., 2015; Tisha et al., 2019). Additionally, the spastic muscle presents structural alterations (e.g., changes in sarcomere length, fiber type, fiber stiffness, stem cell numbers) (Howard and Herzog, 2021) that impact functional deficits such as decreasing force production and limiting the range of motion (Mathewson and Lieber, 2015). Therefore, para-footballers with hypertonia present verifiable coordination problems that impact on the performance of the main physical requirements and football skills evaluated during the classification process, but also presenting differences according to the body area involved and the severity of this eligible impairment (Reina et al., 2020b). The most recent research corpus on evidence-based classification in CP football has explored the relationship between match performance parameters and coordination impairments (Reina et al., 2021b), but also with a battery of activity limitation tests (Reina et al., 2020b). These results have shown a complex interaction between these factors, confirming the need to clarify the impairment-specific associations for classification in para-sport.

At present, classification methods for assessing spastic hypertonia in para-sport are based, principally, on manual testing of overreacting muscle resistance to rapid stretch generated due to increased reflex activity, where it is not possible to differentiate between neural (e.g., spasticity, hyperreflexia) and non-neural (e.g., change of muscle properties) contributors to joint resistance (Dietz and Sinkjaer, 2007; de Gooijer-van de Groep et al., 2013). Considering that earlier investigations described the association between the level of spasticity and sport-specific performance in para-athletes with high support needs of Race Running (van der Linden et al., 2018, 2020), no study has examined the relationship of these type of impairment measures in CP para-athletes with a more functional profile (i.e., ambulatory para-athletes without mobility technical devices). Therefore, this study aimed to assess the association of clinical spasticity measurements with the performance of a sport-specific test (i.e., activity limitation testing) in CP footballers, also considering their impairment profile of spasticity (i.e., unilateral/hemiplegia or bilateral/diplegia).

## Materials and Methods

### Participants

Sixty-nine international male footballers with CP (25.4 ± 7.6 years; 176.3 ± 7.7 cm; 70.1 ± 9.4 kg; 22.6 ± 2.8 kg⋅m^–2^) from 12 national teams of competitors at the World Championships Qualification Tournament (Vejen, Denmark) voluntarily participated in this study. The impairment profile of the participants was 66.7% (*n* = 46) congenital and 33.3% (*n* = 23) with acquired brain injuries. All the participants reported training on average 4.2 ± 1.5 times a week and had 10.1 ± 5.5 years of playing experience. Only para-footballers with a valid license from the International Federation of Cerebral Palsy Football (IFCPF) were involved in this study. Additionally, the inclusion criteria were to have an eligible impairment of spasticity and to be categorized according to their impairment profile of bilateral spasticity (*n* = 15), unilateral spasticity (*n* = 47), and mixed CP profiles (*n* = 7). All participants were classified as level 1 profiles of the Gross Motor Function Classification System (GMFCS) (Jahnsen et al., 2006). As an exclusion criterion, the participants were not injured or rehabilitating from a skeletal muscle injury in the 3 months prior to the time of assessment. Before data collection, participants agreed to participate and gave their informed consent after being informed with a detailed written and oral explanation of the research design.

### Procedures

A cross-sectional design was performed to examine the relationship between lower limb spasticity and physical performance measures. During the classification process, the data were collected a minimum of 72 h before competing in the first official match. According to the CP football classification rulebook, the muscular groups considered to assess spasticity are calf muscles, hamstrings, and adductors due to the several impacts on activity limitation (IFCPF, 2018). Two physiotherapists and official IFCPF classifiers with international experience in Paralympic classification conducted the spasticity assessments before the physical testing session. Before physical testing, a standardized warm-up was performed, followed by activity limitation assessments that had been previously used with CP footballers: dynamic balance [i.e., tandem walk (TW)], coordination [i.e., rapid heel-toe contacts with the dominant (RHT_*D*_) and non-dominant legs (RHT_*ND*_)], vertical and horizontal jump [i.e., countermovement jump (CMJ), standing broad jump (SBJ), triple hop for distance with the dominant (TH_*D*_) and non-dominant legs (TH_*ND*_)], acceleration [i.e., 0–5 m (S0-5m), 5–10 m (S5-10m), and 10–20 m sprint (S10-20m)], and change of direction ability [i.e., modified agility test (MAT) and 505 agility test with ball dribbling (505B)] tests (Reina et al., 2020b). Leg dominance (D: dominant, ND: non-dominant) was considered in accordance with the level of lower limbs impairment for participants with spastic hemiplegia or by the preferred leg for kicking and passing during the game for those with spastic diplegia (Reina et al., 2021a).

### Measures

#### Spasticity Assessment

Spasticity rating measurements were conducted with the Australian Spasticity Assessment Scale (ASAS). This is a simple and standardized testing procedure that combines aspects of the Tardieu Scale and the practical scoring framework of the Modified Ashworth Scale, allowing comparison between muscle groups and categorizing the muscles tested in one category, avoiding ambiguous assessments (Love et al., 2016). In this study, the assessment considered the main group of muscles in joint movements as such as hip adduction (i.e., hip adductors), knee flexion/extension (i.e., hamstring and quadriceps), and ankle plantar flexion (i.e., gastrocnemius and soleus). The assessment was performed on both sides following standardized procedures on an examination bench and was performed once with each player by the two classifiers to obtain a consensus score (Love et al., 2016). According to the catch presence and the degree of resistance generated during the rapid passive stretching movement, a rating ordinal score was assigned to each evaluated muscle group, where 0 indicated no spasticity or catch on rapid passive movement and four affected part(s) rigid contracture segment but moves on slow passive movement (Love et al., 2016). Even though the Modified Ashworth Scale is commonly utilized for both clinical and para-sport settings, the ASAS has been used in Paralympic sports which involve participants with CP, such as Race Running (van der Linden et al., 2020), Boccia (Reina et al., 2015), Para-cycling (Liljedahl et al., 2020), and Para-taekwondo (Para-Taekwondo, 2021). Additionally, ASAS was also utilized in adults with brain injuries (Calame and Singer, 2015), and showed good inter-rater reliability (weighted kappa = 0.87; ICC = 0.88) in the assessment of children with CP (Love et al., 2016). In this study, spasticity scores are reported for the hip (adductors), knee (flexors and extensors) and ankle (dorsiflexors and plantiflexors) for both the more-impaired and less-impaired sides of the body. For the statistical analysis, the muscle spasticity assessment results were summed in each principal joint to provide an overall total score, like previous procedures used in studies of the literature in evidence-based classification and para-sport (van der Linden et al., 2018, 2020).

#### Dynamic Balance: Tandem Walk Test

Participants walked barefoot in a straight line with the front foot placed such that its heel touched the toe of the standing foot to complete a 5 m line as fast as possible and with the best accuracy (Roldan et al., 2020). The trial was not considered valid if the participant lost balance or placed a foot away from the designed straight line. Only one extra attempt was allowed to complete two valid trials with 30 s of rest between attempts (Reina et al., 2021a). The best trial of the test correctly completed in the shortest period (in s) was used for data analysis (Reina et al., 2020b).

#### Coordination: Rapid Heel-Toe Contacts

The participant sat barefoot in full contact with the floor on an adjustable chair (Mobiclinic model Puerto, Seville, Spain). The player was requested to perform 25 cycles at the command, considering that one cycle is the sum of two hits of alternate contacts with the heel and toes separately on a tapping platform (35 cm × 20 cm). The test was performed twice with each leg (RHT_*D*_ and RHT_*ND*_), starting first with the dominant side and heel contact with 1 min rest between attempts. When the participant makes an erroneous contact, the attempt is not considered, and the time (in s) to complete the fastest two trials were registered (Reina et al., 2021b; Sarabia et al., 2021).

#### Vertical Jump: Countermovement Jump

For CMJ, participants were instructed to perform a maximal vertical jump with flexion of the knees during the take-off phase, which was required to be about 90°, and a minimal flexion of the trunk. The hands were placed on the hips during the take-off, flight, and landing phases. Two attempts with rest intervals of 1 min between trials, registering the highest height obtained (in m). Those players who presented spastic hemiplegia and difficulty maintaining their hands on their hips were allowed to keep their hands alongside their body (Yanci et al., 2016; Reina et al., 2018).

#### Horizontal Jump: Standing Broad Jump and Triple Hop for Distance

The SBJ test involved the participants attempting to cover the greatest distance possible with a jump that starts with the legs together and a bilateral take-off/landing. The shortest distance from the landing heel to the starting point was measured (in m). Two attempts with 1 min of rest were permitted, and the best trial was considered for statistical analysis (Reina et al., 2018). In the triple hop (TH) for distance test, the participants performed three consecutive maximal hops and landed on the same leg with the assistance of the arms. The total distance covered was measured from the starting line to the rear of the foot upon final landing. Two attempts were permitted with each foot (TH_*D*_ and TH_*ND*_) with 1 min rest between them, and the best trial with each leg was considered for data analysis (Reina et al., 2018).

#### Acceleration: 0–20 m Sprint

The participants were instructed to accelerate as quickly as possible through a 20 m sprint in a straight line from a standing start with the recording time of 5, 10, and 20 m using infrared photocells (Witty System; Microgate, Bolzano, Italy). Two attempts were permitted with 90 s of rest between attempts, and the fastest trial was used for data analysis (Reina et al., 2020b).

#### Change of Direction Ability: Modified Agility Test and 505 Agility Test With Ball

In the MAT, the participants began behind a starting line in a standing position and performed sprint forward (5-m), following 2.5-m side-stepping toward right, 5-m toward left, 2.5-m toward right again, and completing the course with 5-m backward (Peña-González et al., 2021). The 505B agility test with ball dribbling consisted of running 10-m, sprinting forward to a line 5-m ahead, and pivoting 180° before returning to the 5-m sprint position (Reina et al., 2020b). For both tests, performance (in s) was recorded using infrared photocells (Witty System; Microgate, Bolzano, Italy). Two trials with 2 min of rest were allowed and the fastest time to complete the courses was considered for data analysis.

### Statistical Analysis

This study presents the data as means ± standard deviations (SD) and the mean 95% confidence interval (CI). The normality of the data distribution and homogeneity of variance were assessed using Kolmogorov-Smirnov and Levene’s tests, respectively. The differences between the two trials in each activity limitation tests were determined using Student’s paired *t-*test and, the effect size was given by Cohen’s *d*-values, which were interpreted as follows: above 0.8, between 0.8 and 0.5, between 0.5 and 0.2, and lower than 0.2 were considered large, moderate, small, and trivial, respectively (Cohen, 1988). The reliability among the two trials in each test was assessed with the intra-class correlations (ICC_2,1_) and standard error measurement (SEM). ICC 0.90–1.00 were considered excellent, 0.70–0.89 high, 0.50–0.69 moderate, and < 0.50 as low (Fleiss, 1986). The SEM was calculated by using the formula: *SEM* = $\sqrt{1-\mathrm{ICC}}$. The summation of ordinal scores related to spasticity assessment was used to analyze the association with the performance of activity limitation tests, as was done in previous studies with para-athletes with brain injuries (van der Linden et al., 2018, 2020). Pearson’s correlation coefficient (*r*) analysis was used to examine the relationship between the spasticity scores and the performance in the activity limitation tests. This strength of associations was interpreted as trivial (*r* < 0.1), small (*r* = 0.1–0.3), moderate (*r* = 0.3–0.5), large (*r* = 0.5–0.7), very large (*r* = 0.7–0.9) and nearly perfect (*r* = >0.9) (Hopkins et al., 2009). The statistical analysis was conducted in the Statistical Package for Social Sciences for Windows (version 26.0; SPSS Inc., Chicago, IL, United States). The level of significance was set at *p* < 0.05.

## Results

Table 1 shows the descriptive data of the ASAS spasticity measures in the different joint movements and related muscle groups, also considering the more impaired and non-impaired sides of the body. The minimum spasticity score (i.e., zero) was found for all the single measurements, but a minimum overall spasticity score of 2 was found as minimum impairment criteria to be eligible in CP football. Individual maximum spasticity scores were ranged from 3-to-4, and a maximum overall spasticity score of 30 was obtained for the whole sample. The total score of 30 resulted from the sum of the individual muscle assessments of spasticity during the classification process. Specifically, higher spasticity scores were obtained for the muscles involved in the movement of the distal joints, i.e., ankle muscles had higher ASAS scores than the knee and hip. Also, higher scores of spasticity were found in flexors compared to extensors in both knee and ankle joints.

**TABLE 1:** Descriptive scores from the Australian Spasticity Assessment Scale (ASAS) in muscle groups and total score by all para-footballers.

ASAS score	More-impaired side			Less or non-impaired side			Overall score		
	Min	Max	Mean (SD)	Min	Max	Mean (SD)	Min	Max	Mean (SD)
Hip adductors	0	3	1.00 ± 1.00	0	3	0.32 ± 0.76	0	6	1.29 ± 1.53
Knee (combined)	0	7	2.75 ± 1.92	0	6	0.49 ± 1.26	0	12	3.25 ± 2.54
Extensors	0	3	1.12 ± 1.23	0	3	0.17 ± 0.59	0	6	1.29 ± 1.52
Flexors	0	4	1.64 ± 1.07	0	3	0.32 ± 0.76	0	6	1.96 ± 1.45
Ankle (combined)	0	8	3.33 ± 2.39	0	8	0.88 ± 1.78	0	16	4.25 ± 3.43
Dorsiflexors	0	4	1.30 ± 1.58	0	4	0.29 ± 0.89	0	8	1.59 ± 2.10
Plantiflexors	0	4	2.03 ± 1.26	0	4	0.59 ± 1.05	0	8	2.62 ± 1.70
Total score	0	24	7.20 ± 6.79	0	15	1.70 ± 3.52	2	30	8.78 ± 6.24

*Min, minimum; Max, maximum; SD, Standard deviation.*

Descriptive results and within-session reliability for each physical test (i.e., dynamic balance, coordination, vertical and horizontal jumps, acceleration and change of direction ability) in all footballers with CP are presented in Table 2. No significant differences were found among the two trials performed in each activity limitation test, with trivial to small effect sizes (*p* > 0.05, *d* = 0.00 to 0.35). The reliability of TH_*D*_, TH_*ND*_, and S10-20m was categorized as excellent [ICC_2,1_ = 0.91 to 0.93 (0.86–0.96); SEM = 0.11 to 0.31]. A high ICC was obtained for tandem walk, countermovement jump, standing broad jump, sprint 0–5 m, modified agility test, 505 with ball, and rapid heel-toe contacts with dominant and non-dominant legs [ICC_2,1_ = 0.71 to 0.89 (0.54–0.93); SEM = 0.02 to 2.61]. However, S5-10m presented low reliability scores [ICC_2,1_ = 0.44 (0.16–0.63); SEM = 0.19].

**TABLE 2:** Descriptive activity limitation test results (mean ± standard deviation), comparison for each trial and intra-class correlation by all para-footballers.

	M ± SD	95 % CI	Trial 1 M ± SD	Trial 2 M ± SD	ES	ICC_2,1_ (95% CI)	SEM
**Dynamic Balance**							
TW (s)	21.1 ± 7.8	18.8 to 23.3	22.0 ± 8.2	20.2 ± 7.5	0.22	0.89 (0.83–0.93)	2.61
**Coordination**							
RHT_*D*_ (s)	7.82 ± 1.81	7.06 to 8.59	10.02 ± 3.43	8.82 ± 3.17	0.35	0.80 (0.72–0.86)	1.48
RHT_*ND*_ (s)	10.60 ± 3.03	9.32 to 11.88	12.08 ± 4.82	11.26 ± 4.27	0.17	0.89 (0.85–0.93)	1.49
**Vertical Jump**							
CMJ (m)	0.44 ± 0.07	0.41 to 0.47	0.41 ± 0.06	0.42 ± 0.06	−0.17	0.89 (0.83–0.92)	0.02
**Horizontal Jump**							
SBJ (m)	1.80 ± 0.27	1.68 to 1.91	1.55 ± 0.3	1.62 ± 0.3	−0.23	0.81 (0.72–0.87)	0.13
TH_*D*_ (m)	5.08 ± 1.04	4.64 to 5.52	4.44 ± 1.05	4.66 ± 1.08	−0.21	0.92 (0.87–0.95)	0.31
TH_*ND*_ (m)	3.18 ± 1.23	2.66 to 3.70	3.03 ± 1.0	3.21 ± 1.07	−0.18	0.91 (0.86–0.95)	0.31
**Acceleration/Sprint**							
S0-5m (s)	0.85 ± 0.09	0.81 to 0.89	0.9 ± 0.1	0.9 ± 0.1	0.00	0.71 (0.54–0.82)	0.07
S5-10m (s)	1.65 ± 0.26	1.54 to 1.76	1.75 ± 0.27	1.77 ± 0.22	−0.07	0.44 (0.16–0.63)	0.19
S10-20m (s)	3.11 ± 0.42	2.93 to 3.29	3.33 ± 0.32	3.25 ± 0.45	0.25	0.93 (0.87–0.96)	0.11
**Change of Direction Ability**							
MAT (s)	7.33 ± 1.14	6.85 to 7.81	7.87 ± 1.37	7.51 ± 0.96	0.26	0.82 (0.73–0.88)	0.51
505B (s)	2.72 ± 0.45	2.53 to 2.91	2.9 ± 0.57	2.85 ± 0.4	0.09	0.74 (0.61–0.83)	0.25

*M, mean; SD, standard deviation; CI, confidence interval; ES, effect size; ICC, intra-class correlations; SEM, standard error measurement; TW, tandem walk; RHT_D_, rapid heel-toe dominant leg; RHT_ND_, rapid heel-toe non-dominant leg; CMJ, countermovement jump; SBJ, standing broad jump; TH_D_, triple hop for distance dominant leg; TH_ND_, triple hop for distance non-dominant leg; S0-5m, sprint 5m; S5–10m, sprint 10m; S10–20m, sprint 20 m; MAT, modified agility test; 505B, 505 test with ball.*

**FIGURE 1:**
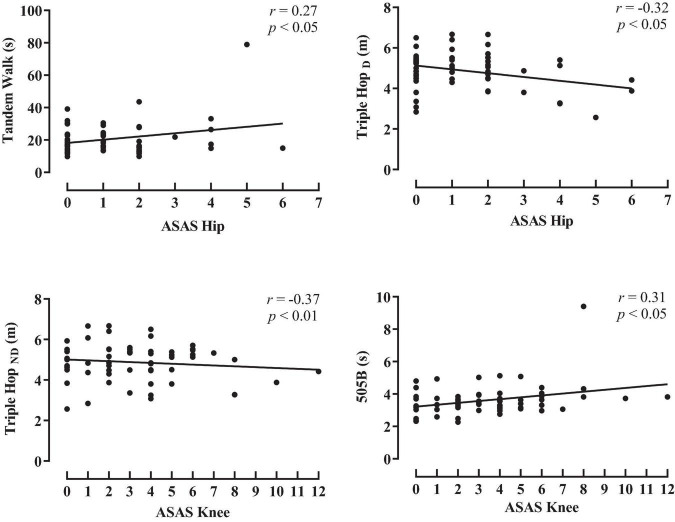
Pearson product correlation between scores for the Australian Spasticity Assessment Scale (ASAS) in hip and knee muscle groups and activity limitation test performance by all para-footballers. TW, tandem walk, TH_*D*_, triple hop for distance dominant leg; TH_*ND*_, triple hop for distance non-dominant leg; 505B, 505 agility test with ball.

**FIGURE 2:**
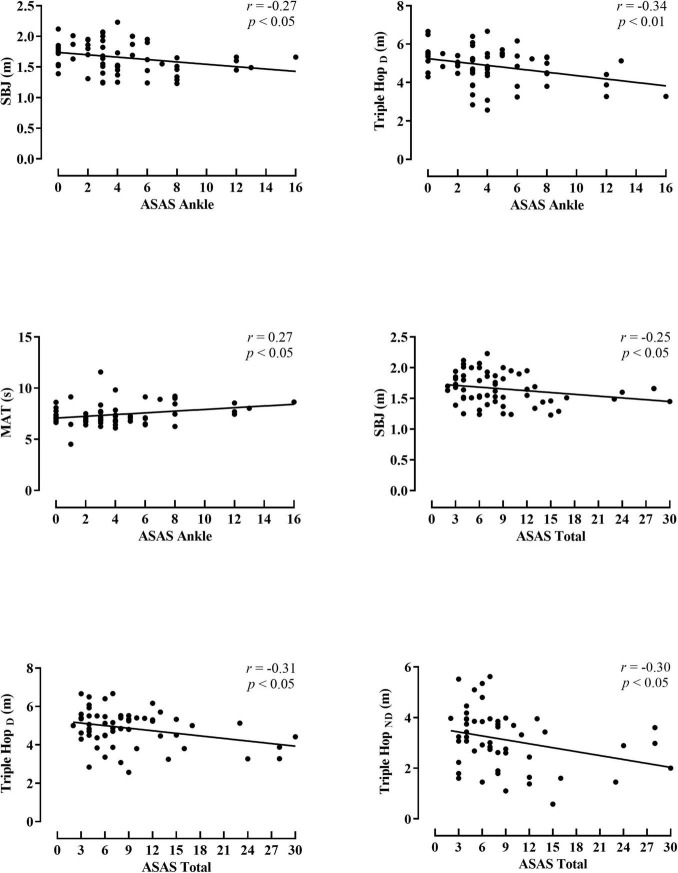
Pearson product correlation between scores for the Australian Spasticity Assessment Scale (ASAS) in ankle muscle group, and total score with activity limitation test performance by all para-footballers. SBJ, standing broad jump; TH_*D*_, triple hop for distance dominant leg; TH_*ND*_, triple hop for distance non-dominant leg; MAT, modified agility test.

The associations between the spasticity scores and the activity limitation variables are shown in Figures 1, 2 (hip and knee) (ankle and total spasticity scores). Trivial to moderate relationships were described between spasticity scores and activity limitation tests by overall participants. A significant correlation was found among hip spasticity scores, tandem walk, and TH_*D*_ (*r* = 0.27 to −0.32, *p* < 0.05, small-to-moderate). Moreover, players with higher knee spasticity scores demonstrated lower performance in the TH_*ND*_ and 505 with ball test (*r* = 0.31 to −0.37, *p* < 0.05, moderate). Similarly, a significant relationship was obtained between ankle spasticity scores and performance in the standing broad jump (*r* = −0.27, *p* < 0.05, small), TH_*D*_ (*r* = −0.34, *p* < 0.01, moderate), and modified agility test (*r* = 0.27, *p* < 0.05, small). Considering the total spasticity scores, players with better horizontal performance apparently presented a lower presence of spasticity, considering the assessments of standing broad jump, TH_*D*_, and TH_*ND*_ (*r* = −0.25 to −0.31, *p* < 0.05, small-to-moderate).

## Discussion

The aim of this study was to examine the association between spasticity and sports-specific performance measurements in international footballers with CP. This research found small-to-moderate associations between the level of spasticity in the lower limbs and the measures of coordination, horizontal jumps, and change of direction ability performance, where performance may decrease as the muscle spasticity score increases. In the context of Paralympic classification, identifying the relative strength of association between impairment measures and sport-specific determinants of performance is a mandatory step to develop evidence-based classification systems (Connick et al., 2018). To the authors’ best knowledge, this is the first study to analyze the associations between spasticity impairment scores and performance (i.e., activity limitation) testing in a team para-sport for ambulant para-athletes with CP.

Previous investigations have attempted to examine the predictive value of impairment measures in para-sport performance but using *ad hoc* measurements of impaired coordination in different Paralympic sports. In this line, Connick et al. (2015) examined the extent to which range of movement and coordination impacts running performance in para-athletes with brain injuries, demonstrating no significant association between (impaired) lower limb coordination and sprint performance. Subsequently, Beckman et al. (2016) explored the relationships between (impaired) strength and running performance in 13 participants with eligible impairments of hypertonia, athetosis and, ataxia, but they did not find significant associations between these measurements. However, other studies with para-athletes with more severe impairment related to brain injuries reported significant associations among spasticity scores and speed in Race Running, suggesting the impact of the impairment on the sport-specific performance (van der Linden et al., 2018, 2020). The significant strength of the association (i.e., trivial-to-moderate) between spasticity and the physical tests obtained in the present study suggests a low impact of lower limbs’ spasticity on sports performance in para-footballers with a high level of proficiency (i.e., international-level and GMFCS Level 1). Also, the ordinal nature of the current clinical assessments of spasticity (i.e., scales from 0 to 4 scores) constrains the establishment of evidence-based relationships between valid and reliable measurements of impairment and sports performance (Tweedy et al., 2014). Furthermore, this challenge is in line with the findings of Chiu et al. (2011), who described that spasticity contributes to only 9% of motor impairments, along with coordination (21%), contracture (7%), and strength (1%) in people with CP. However, spasticity clinical measures are extensively used in different para-sport disciplines (e.g., para-athletics, para-swimming, boccia, para-table tennis, wheelchair rugby, para-taekwondo) and are performed under strict protocols with the goal of identifying the level of impairment (van der Linden et al., 2018, 2020).

Hip adductor spasticity has been described as a negative component that impacts body motion and could induce pathological characteristics reflected in a limited range of movement of joints and motor dysfunctions (i.e., in people with severe hip dislocation impairments; Larkin-Kaiser et al., 2019). The characteristics of adductor muscle spasticity may influence the activities that require dynamic balance with unilateral pedal stance in the lower extremities, such as the above-mentioned assessments. The results of this study indicate that higher scores of hip adductors spasticity could affect the main muscular groups necessary to perform the tandem walk (i.e., dynamic balance) and TH tests (i.e., unilateral jumping), attributable to the possible negative interference in voluntary movements due to neural and non-neural related characteristics of the upper motor neuron lesion (Bar-On et al., 2015). Dynamic balance estimated with the tandem walk test has been previously used in studies with CP footballers and demonstrates an altered execution that could also be related to sports performance (Reina et al., 2020b,2021a). The findings of this study reinforce the possibility that spasticity, in conjunction with involuntary movements, alterations of co-contractions, lack of control in muscle patterns activation, and compensatory strategies, could impact on dynamic balance, and so in the mechanical efficiency in ambulation activities of adults with CP (Dietz and Sinkjaer, 2007; Bar-Haim et al., 2013). In the case of the TH test, hip musculature contributes to the horizontal displacements where the performance presents strong associations with sprint in non-impaired footballers (Maulder and Cronin, 2005) and is proposed as a valid tool for classification purposes (Reina et al., 2018). Although the associations found in this study were low-to-moderate, it appears that the presence of spasticity in hip adductors muscle groups, which causes velocity-dependent resistance and contributes to abnormal synergies and disordered motor control, could affect the total distance in the horizontal jump due to the higher intersegmental coordination and stability requirements to perform these movement patterns (Sanger et al., 2003; Reina et al., 2018; Li et al., 2019).

Regarding the spasticity affecting the muscle groups involved in the motion of the knees, higher scores in flexors were found compared to knee extensors, in line with other clinical studies with CP participants with similar GMFCS profiles (Pierce et al., 2008). The ASAS scores showed a significant association of the horizontal jump with the unilateral non-dominant lower limb (TH_*ND*_) and the change of direction ability in the 505 with ball test. Likewise, Reina et al. (2020b) reported a significant relationship of TH performance with the maximum velocity and the number of high accelerations during matches in para-footballers with bilateral spasticity and other functional profiles (including spastic hemiplegia), respectively. In this regard, considering horizontal force production during motor activities, the hamstring musculature plays a determinant role in the contribution of sprint mechanical properties and sprint acceleration performance in non-disabled athletes (Morin et al., 2015; Nuell et al., 2021). Due to the implications of knee joint musculature during horizontal jumps, it is plausible to think that spasticity impairment could affect the performance of those movements that require higher balance and coordination demands (Reina et al., 2018). Moreover, taking into account that muscular spasticity is characterized by changes in passive mechanical properties, which limit the range of motion permissible at the joints and have consequences on active force generation, these factors may have an impact on motor capabilities affecting the performance of an activity limitation test in CP footballers (Dayanidhi and Lieber, 2018). On the other hand, it has been demonstrated that change of direction actions have high eccentric requirements, especially in the knee flexors and extensors, necessary to perform maneuvers in technical and tactical player movements in the course of the match (Sheppard and Young, 2006; Chaouachi et al., 2012). Specifically, the change of direction could be related with spasticity scores as this type of test forces the muscle to overreact into a rapid stretch (Graham et al., 2016), influencing the capacity to perform activities that require quick accelerations and decelerations of the body (Chaabene et al., 2018), which is frequently observed in CP football matches (Yanci et al., 2018). In such a case, it could be plausible to suggest that the 505B was a more demanding task due to the ball movement activity than the modified agility test, where spasticity could play a role during the intention to maintain ball control and perform a rapid change of direction. Some caution needs to be considered for the interpretation of these results because no significant associations were found between knee spasticity scores and the other tests, possibly because spasticity could be masked by the muscle activation required in motor demanding activities (van der Krogt et al., 2010).

Concerning the ankle region, plantiflexors exhibited higher spasticity scores compared to dorsiflexors, constraining the range of movement of the ankle (Hägglund and Wagner, 2011). It is well-known that the effects of the brain lesion associated with changes in functional and structural properties of the spastic muscle may extend to different components of the musculoskeletal system, producing abnormal postures such as ankle forced to the equinus position, resulting in energy-inefficient gait patterns (Graham et al., 2016). In this study, ankle muscle group spasticity scores presented a significant negative relationship with horizontal jump tests (e.g., standing broad jump and TH_*D*_) and change of direction ability (e.g., modified agility test), showing the negative impact of impairment on jumping and change of direction performance, respectively. Although no significant associations were found with sprint performance in this study, the soleus and gastrocnemius muscles have been described as the principal contributors to forward propulsion and support during running performance (Hamner et al., 2010). Indeed, a recent study indicated that para-athletes with brain injuries compensate for motor impairments with particular characteristics such as reduced stride length, step length, flight time, and increased contact time during sprinting, and running performance (Fiorese et al., 2020). Additionally, de Gooijer-van de Groep et al. (2013) reported that CP adolescents, compared to a non-impaired age-matched group, presented a larger contribution of neural components to ankle joint stiffness using a determined assessment technique. However, contrary to this, Geertsen et al. (2015) suggested that impaired gait function in adults with CP is more likely due to reduced rapid force generation and increased passive stiffness rather than spasticity in ankle muscles. Possibly, the mechanical differences that components present in the spastic muscle, such as overstretched sarcomeres, reduced muscle size and decreased contractile tissue (Howard and Herzog, 2021) impact more on the capacity to jump horizontally than in sprinting due to the higher intersegmental coordination, balance and stability demands to stabilize the body during the braking phase (Reina et al., 2018). Hence, more research is necessary to elucidate the impact of neural and non-neural components of hypertonia on specific activity limitations associated with sports performance.

Cerebral palsy is a condition with many features and phenotypic variations, where to investigate the relationship between impairment and activity limitation could represent a considerable scientific challenge (Graham et al., 2016; Reina et al., 2020b). Additionally, exploring the impact of muscle weakness and restricted range of motion in key joint determinants on performance in football could result in a deeper understanding of these variables, which interact with the eligible impairments determined by the International Paralympic Committee, and specifically by CP-Football (i.e., hypertonia, ataxia or athetosis). However, some additional study limitations should be mentioned. First, although the use of clinical scales for assessing spasticity in para-athletes is an extended practice in Paralympic sport, this is based on the screening of joint resistance mediated by passive velocity-dependent mobilization, where the fundamental problem is the oversimplification of neural and non-neural components contributions to the resistance to muscle stretch (de Gooijer-van de Groep et al., 2013). Further studies should consider biomechanical and electrophysiological methods that permit identifying under dynamic conditions the impact of spasticity on sport activity limitation tests necessary for player class allocation (Geertsen et al., 2015). Also, sport-specific ranges of motion should be considered for a better understanding how spasticity may affect sports performance. Second, the overrepresentation of para-footballers with unilateral hemiplegia in CP football (Reina et al., 2020a) occurred in this study, with unequal groups for between-groups comparisons according to the impairment profile. Additionally, the assessment of spasticity might be biased by inter-observer (i.e., classifier) reliability or other contextual factors such as fatigue, temperature, or non-reported botulin toxin treatments. The ordinal nature of this measurement and the recommendations for developing an evidence-based classification system (Tweedy et al., 2014, 2018), alternative, and/or additional assessment methods of this eligible impairment in Paralympic sport (i.e., hypertonia) are required, also considering the weak associations found with ambulant athletes with CP or brain injury (Connick et al., 2015; Beckman et al., 2016; Reina et al., 2021b). In addition, future studies would consider other spasticity measurements such as the Modified Ashworth scale (Meseguer-Henarejos et al., 2018) due to its common use in clinical practice but also in other Paralympic sports such as para-athletics or para-swimming, among others. Finally, although good reliability scores were found when comparing trials 1 and 2 for all the activity limitation tests, demonstrating para-athletes consistency in their performance (i.e., no learning or fatigue), the multiple *t*-test used for this study (i.e., one per activity limitation test) should be considered in further analyses that include all those tests (e.g., correction factor). However, this study provides specific reliability scores for CP footballers with unique eligible impairments of hypertonia (i.e., spasticity) because recent research suggested a potential impairment-specific relationship between eligible impairment and para-sports performance (Reina et al., 2020b).

## Conclusion

This study suggests that the amount of spasticity according to each evaluated joint muscle group of the lower limbs presents a low-to-moderate significant relationship with determined measures of dynamic balance, coordination, horizontal jump, acceleration, and change of direction ability with and without the ball in international-level CP footballers. While hypertonia is described as an international standard of eligible impairment in Paralympic sport (International Paralympic Committee, 2016), this is currently measured by clinical assessment methods of spasticity. Hence, the contribution of spasticity to hypertonia should be better described for eligibility in Paralympic sport and needs to be more specific considering the neural and non-neural factors that impact motor activity performance. As CP football presents complex physical and technical demands, this study provides some evidence concerning the negative impact of spasticity when performing relevant motor-specific skills.

## Data Availability Statement

The original contributions presented in the study are included in the article/Supplementary Material, further inquiries can be directed to the corresponding authors.

## Ethics Statement

The studies involving human participants were reviewed and approved by The Institutional Review Committee of Miguel Hernández University approved the protocol study (Reference no. DPS.RRV.01.14). All the participants provided their written informed consent to participate in this study. The patients/participants provided their written informed consent to participate in this study.

## Author Contributions

AR and RR: conceptualization, methodology, and visualization. RR and DC: formal analysis. AI, DC, and MH: investigation. RR and JY: resources and supervision. RR and MH: data curation. AR, RR, MH, and JY: writing—original draft preparation. RR, AR, DC, AI, JY, and MH: writing—review and editing. RR: project administration and funding acquisition. All authors have read and agreed to the published version of the manuscript.

## Conflict of Interest

The authors declare that the research was conducted in the absence of any commercial or financial relationships that could be construed as a potential conflict of interest.

## Publisher’s Note

All claims expressed in this article are solely those of the authors and do not necessarily represent those of their affiliated organizations, or those of the publisher, the editors and the reviewers. Any product that may be evaluated in this article, or claim that may be made by its manufacturer, is not guaranteed or endorsed by the publisher.
